# Application of Feedback System Control Optimization Technique in Combined Use of Dual Antiplatelet Therapy and Herbal Medicines

**DOI:** 10.3389/fphys.2018.00491

**Published:** 2018-05-04

**Authors:** Wang Liu, Yu-Long Li, Mu-Ting Feng, Yu-Wei Zhao, Xianting Ding, Ben He, Xuan Liu

**Affiliations:** ^1^Institute of Interdisciplinary Integrative Biomedical Research, Shanghai University of Traditional Chinese Medicine, Shanghai, China; ^2^Institute for Personalized Medicine, State Key Laboratory of Oncogenes and Related Genes, School of Biomedical Engineering, Shanghai Jiao Tong University, Shanghai, China; ^3^Department of Cardiology, Ren Ji Hospital, School of Medicine, Shanghai Jiao Tong University, Shanghai, China

**Keywords:** feedback system control, dual antiplatelet therapy, herbal medicine, platelet aggregation, optimization, synergism

## Abstract

**Aim:** Combined use of herbal medicines in patients underwent dual antiplatelet therapy (DAPT) might cause bleeding or thrombosis because herbal medicines with anti-platelet activities may exhibit interactions with DAPT. In this study, we tried to use a feedback system control (FSC) optimization technique to optimize dose strategy and clarify possible interactions in combined use of DAPT and herbal medicines.

**Methods:** Herbal medicines with reported anti-platelet activities were selected by searching related references in Pubmed. Experimental anti-platelet activities of representative compounds originated from these herbal medicines were investigated using *in vitro* assay, namely ADP-induced aggregation of rat platelet-rich-plasma. FSC scheme hybridized artificial intelligence calculation and bench experiments to iteratively optimize 4-drug combination and 2-drug combination from these drug candidates.

**Results:** Totally 68 herbal medicines were reported to have anti-platelet activities. In the present study, 7 representative compounds from these herbal medicines were selected to study combinatorial drug optimization together with DAPT, i.e., aspirin and ticagrelor. FSC technique first down-selected 9 drug candidates to the most significant 5 drugs. Then, FSC further secured 4 drugs in the optimal combination, including aspirin, ticagrelor, ferulic acid from DangGui, and forskolin from MaoHouQiaoRuiHua. Finally, FSC quantitatively estimated the possible interactions between aspirin:ticagrelor, aspirin:ferulic acid, ticagrelor:forskolin, and ferulic acid:forskolin. The estimation was further verified by experimentally determined Combination Index (CI) values.

**Conclusion:** Results of the present study suggested that FSC optimization technique could be used in optimization of anti-platelet drug combinations and might be helpful in designing personal anti-platelet therapy strategy. Furthermore, FSC analysis could also identify interactions between different drugs which might provide useful information for research of signal cascades in platelet.

## Introduction

Anti-platelet therapy is an essential component of treatment in patients with coronary artery disease, especially those underwent percutaneous coronary intervention (Abbott, 2017; Majithia and Bhatt, 2017). Dual antiplatelet therapy (DAPT), a combination of aspirin and P2Y12 receptor antagonist, clopidogrel or ticagrelor, is popularly used in clinic. Inactivation of cyclooxygenase by aspirin and concomitant blockade of the P2Y12 receptor on the platelet surface by reversible or irreversible receptor antagonists could effectively reduce platelet aggregation and the risk of vascular occlusion (i.e., stent thrombosis) (Binder and Lüscher, 2015). Generally, for patients underwent percutaneous coronary intervention, an arbitrary recommendation for 12 months of DAPT after drug eluting stent implantation was issued by Cardiology Guideline Committees (Roffi et al., 2016). For these patients who have to take at least 12 months of DAPT, the fine-tuning of platelet activation and inhibition is very important because either bleeding or thrombosis would cause severe events in clinic. Therefore, popularly used herbal medicines and/or food additives with possible anti-platelet activities should be taken as a factor which could not be ignored.

Herbal medicines such as traditional Chinese medicine (TCM) are popularly used in China as well as in other Asian countries. According to a survey, more than 71.2% patients adopted integration of TCM and western medicine (conventional drugs) in clinic in China (Chen and Lu, 2006). Especially, in a real word study of 84,697 patients with coronary heart disease, 43.46% of patients in anti-platelet therapy also took TCMs with anti-platelet effects at the same time (Li et al., 2014). In Western countries, herbal medicine use is also growing. Preparations including single herb preparations, ethnic and modern herbal medicine formulations are widely used as adjunct therapies or to improve consumer wellbeing (Barnes et al., 2016; Job et al., 2016; Sammons et al., 2016; Teng et al., 2016; Enioutina et al., 2017). On one hand, herbal medicine interaction with conventional drugs such as DAPT may result in inadequate dosing of DAPT or adverse reactions and cause serious problems (Enioutina et al., 2017). While, on the other hand, combined use of herbal medicines with DAPT might be used as a method to optimize DAPT. To be noted, some patients underwent DAPT therapy could still experience ischemic events and lots of efforts to optimize DAPT were conducted (Binder and Lüscher, 2015; Palmerini and Stone, 2016; Sheyin et al., 2016; Verdoia et al., 2016; Paravattil and Elewa, 2017). Therefore, it is necessary and important to optimize dose strategy of combinations of herbal medicines and DAPT and to clarify the interactions between different drugs in the combinations.

**Figure 1:**
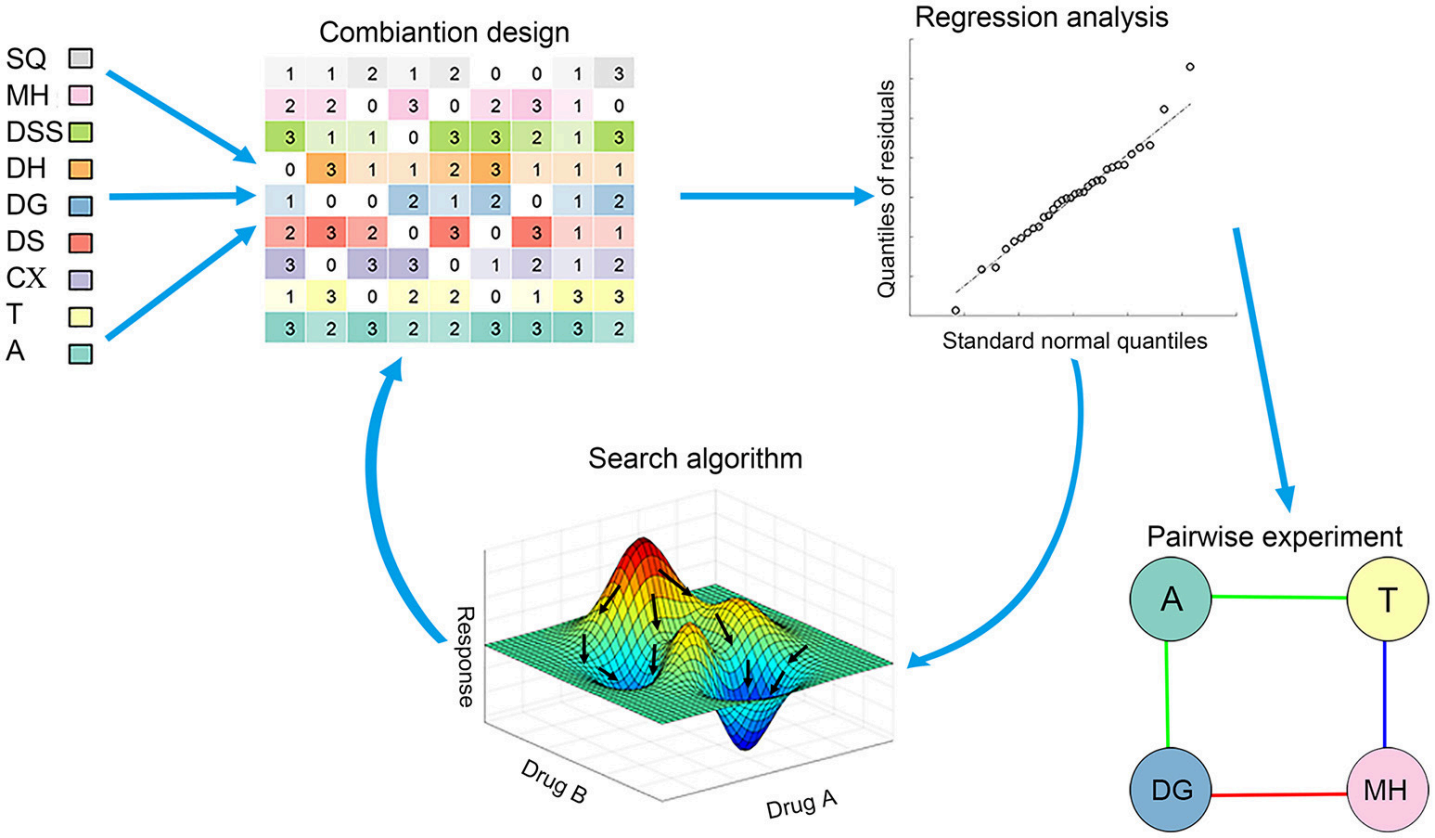
Scheme of the FSC application in this study. The optimization process is based on an iterative cycle, in each generation drug combinations were tested *in vitro* and analyzed with mathematical models. The feedback search algorithm therefore uses the difference between the results of the tested combinations and the desired optimization goals to predict new combinations to be tested in the next generation. After the optimization process, we conducted a pairwise experiment to confirm conclusion from the FSC optimization process.

Feedback system control (FSC) (Nowak-Sliwinska et al., 2016) is a recently developed technique which has been widely used in the optimization of drug combinations. The optimization procedure of FSC is phenotypically driven and does not require any mechanistic information of the system. Thus, FSC can be successfully applied in various complex biological systems (Weiss et al., 2015; Ding et al., 2017; Lee et al., 2017). Herein, for the first time, FSC technique is applied in the study of anti-platelet drug combinations. In the present study, we adopted FSC technique to optimize the combination of DAPT and herbal medicines in anti-platelet therapy. Scheme of the FSC application in this study is shown in Figure 1. With two generations of FSC optimization, the optimized drug combination and potent interactions between drug pairs were predicted. Then, the predicted interactions between drug pairs were further confirmed using Combination Index (CI) analysis (Chou, 2010).

## Materials and methods

### Materials

Adenosine 5′-diphosphate (ADP), aspirin and ticagrelor were obtained from Sigma-Aldrich (St Louis, MO, USA). Ligustrazine, salvianolic acid B, 5-hydroxymethylfurfuraldehyde, garlicin and panax notoginsenosides were purchased from Shanghai Nature Standard Biotechnology Co., Ltd. (Shanghai, P. R. China). Forskolin and ferulic acid were purchased from Dalian Meilun Biotech Co., Ltd. (Dalian, P.R. China).

### Making list of herb medicines with anti-platelet activities

References reported anti-platelet activities of herbal medicines, mainly TCM, were searched in Pubmed by using “traditional Chinese medicine” and “platelet” as key words. Then, the references were checked individually to record the information of herbs with anti-platelet activities. Herbs were then listed according to the frequency of reports, i.e., numbers of references which reported the anti-platelet activities of the herb.

### Preparation of rat platelet-rich-plasma

Male Sprague-Dawley rats (230–250 g) were obtained from SLAC Laboratory Animal Co., Ltd. (Shanghai, P.R. China). Animal Experimental study was carried out in accordance with the recommendations of National Institutes of Health Guidelines on the Use of Laboratory Animals. The protocol was approved by the Committee on the Ethics of Animal Experiments of the School of Medicine, Shanghai Jiao Tong University. Blood was taken from anesthetic rat by direct puncture of the abdominal aorta with a 21-gauge needle and transferred into plastic tubes containing 3.8% trisodium citrate as anticoagulant in a volume ratio 9:1. Platelet-rich plasma (PRP) was obtained by centrifugation of blood at 200 × g for 10 min at room temperature.

### Checking the anti-platelet activities of drugs

The anti-platelet activities of drugs were experimentally determined by checking their inhibiting effects on ADP-induced platelet aggregation of rat PRP. Generally, drugs were dissolved in natural saline. For the drugs such as aspirin which could not be well dissolved in natural saline, drugs would be dissolved in ethanol and then be serially diluted in natural saline to keep the final concentration of ethanol in the assay system to be less than 0.1%. Rat PRP was incubated with various concentrations of tested drugs or vehicle control (natural saline or natural saline with 0.1% ethanol) at 37°C for 5 min before induction of platelet aggregation. Then, platelet aggregation was induced as reported in our previous papers (Yao et al., 2008a,b; Ma et al., 2011). Briefly, PRP was stimulated with 10 μM ADP under continuous stirring at 37°C in a silicone-treated glass cuvette and recorded using an aggregometer (Model TYXN-96, TongYong Corp., Shanghai, China). The inhibitory effect of treatments on platelet aggregation was expressed as the percentage of inhibition relative to the control using the following equation:

$$\begin{array}{l} \text{ Inhibitory effect }(\%)\\ =\left(1-\frac{\text{aggregation percentage of treatment}}{\text{aggregation percentage of control sample}}\right)\times 100\% \end{array}$$

The IC50 value (half-maximal inhibitory concentration) was calculated based on nonlinear fit of the log values using GraphPad Prism® 5 (Version 5.01, GraphPad Software, Inc., USA).

### Neural networks models

The protocol of applying the FSC approach was described in previous literatures (Li et al., 2016; Nowak-Sliwinska et al., 2016). Herein, FSC used a single hidden layer two-neuron multilayer perceptron to fit the data in generation 1. The input is the coded dose level in the drug combination, and output is anti-platelet efficacy of the combination. The network was constructed and trained using the neuralnet (Günther and Fritsch, 2010) R package.

### Linear regression model

FSC scheme applied Linear regression model to fit the data in generation 2. The Regression analysis was performed based on interaction model and quadratic model with the following forms:

$$\begin{array}{l} \;y=\beta_{0}+\sum_{i\;=\;1}^{k}\beta_{i}x_{i}+\sum_{i\;=\;1}^{k}\sum_{j\;=\;i+1}^{k}\beta_{ij}x_{i}x_{j}+\varepsilon \\ y=\beta_{0}+\sum_{i\;=\;1}^{k}\beta_{i}x_{i}+\sum_{i\;=\;1}^{k}\sum_{j\;=\;i+1}^{k}\beta_{ij}x_{i}x_{j}+\sum_{i\;=\;1}^{k}\beta_{ii}x_{i}{}^{2}+\varepsilon \end{array}$$

Where β_0_, β_*i*_, β_*ii*_, and β_*ij*_ are the intercept, linear quadratic and interaction terms, respectively; *y* is the response variable (i.e., anti-platelet efficacy); *x*_*i*_ and *x*_*j*_ are independent variables (i.e., coded drug dose level); ε is an noise term with zero mean.

### Analysis of interactions in drug pairs

To confirm predicted possible interactions in drug pairs, we used constant ratio design to quantitatively measure the degree of drug interaction in terms of synergism and antagonism. We conducted 4 drug pair experiments, in each drug pair experiment two drugs were combined with different concentrations while the concentration ratio between them was held at constant. A series of concentrations (at least 5) of each drug pair were designed and then the experimental anti-platelet activities of the drug pairs were checked. CI calculation (Chou, 2006, 2010) was applied to measure the drug combination interaction. The CI theory of Chou-Talalay offers quantitative definition for additive effect (CI = 1), synergism (CI < 1), and antagonism (CI > 1) in drug combinations.

### Statistical analysis

In results of experimental checking anti-platelet activities of individual drugs, data are expressed as mean ± SEM of three independent experiments. The statistical analysis for the modeling, including Cook's distance and residual analysis, is provided in Supplemental Figures 1, 2. Drug combination synergy between drug pairs was quantified using CI analysis (Chou, 2006, 2010). The CI value calculated for each drug pair indicated synergistic interaction (CI < 1), additive effect (CI = 1) or antagonism (CI > 1).

## Results

### List of herb medicines with reported anti-platelet activities

In our searching of published papers in Pubmed using keywords of “traditional Chinese medicine” and “platelet,” there were 556 related articles up to September 8, 2016. By checking the references individually and manually, 67 herbs with reported anti-platelet activities were found. Based on our previous research results, *Coleus forskohlii(Wild.)Briq*., a herb medicine popularly used but not categorized as TCM, was also included. The herb medicines were then summarized and listed according to the number of references reported their anti-platelet activities (Supplemental Table 1).

### Experimental anti-platelet activities of drugs

**Figure 2:**
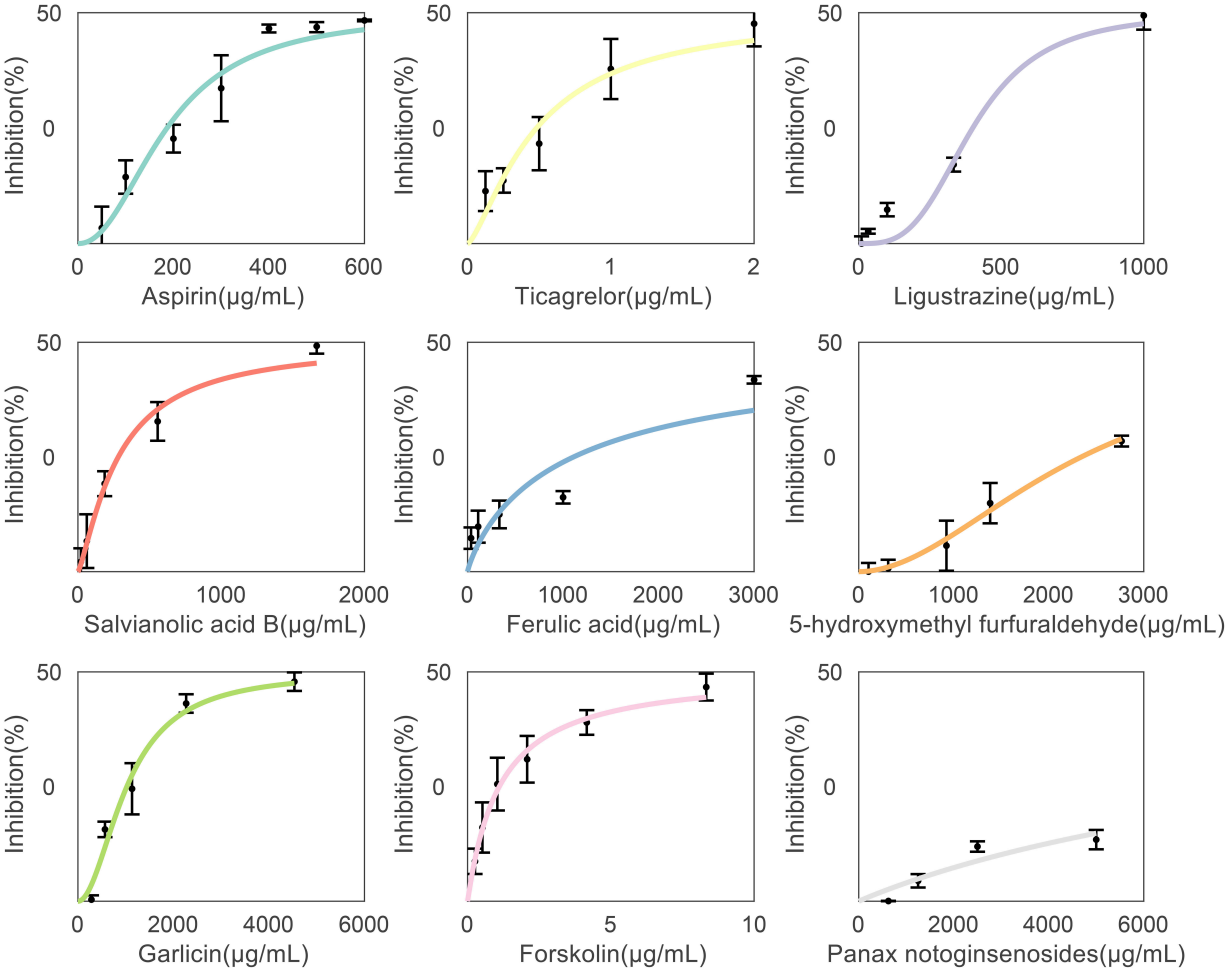
Individual drug dose response curves. The anti-platelet activities of drugs were determined by checking their inhibiting effects on *in vitro* ADP-induced platelet aggregation of rat PRP. Shown were the experimental single drug dose response curves for the nine drugs used in this study. The curves were fitted using Hill equations, and the data was used to identify the drug concentrations to be used in combination studies.

By using the *in vitro* ADP-induced PRP aggregation assay, anti-platelet activities of aspirin, ticagrelor and representative components of herbal medicines listed in Supplemental Table 1 were examined. While, many herbal components showed weak or only moderate anti-platelet potency, which made it impossible to calculate their IC50 values in inhibiting platelet aggregation. For example, panax notoginsenosides, which is main components of SanQi, exhibited only about 20–25% inhibition rate at doses more than 2,500 μg/mL. Generally, FSC analysis was conducted based on drugs with experimentally determined IC50 values. Anyway, since the present work is the first study trying to conduct FSC analysis for anti-platelet herbal components, panax notoginsenosides was also included in the analysis system as a trial. As shown in Table 1, Figure 2, the anti-platelet activities of individual drugs were observed. IC50 values of aspirin, ticagrelor and 6 herbal components (Ligustrazine from ChuanXiong, Salvianolic acid B from DanShen, Ferulic acid from DangGui, Forskolin from MaHouQiaoRuiHua, 5-hydroxymethyl furfuraldehyde from DiHuang, Garlicin from DaSuan) were shown in Table 1. Anti-platelet activity of Panax notoginsenosides from SanQi was also described in Table 1. The dose-effect curves of the drugs were shown in Figure 2.

**Table 1:** Anti-platelet activities of aspirin, ticagrelor and herb components used in FSC analysis.

**Name**	**Related herb**	**Abbr**.	**IC50 value (μg/mL)**
Aspirin	–	A	187.3 ± 18.96
Ticagrelor	–	T	0.505 ± 0.0992
Ligustrazine	ChuanXiong	CX	388.9 ± 21.69
Salvianolic acid B	DanShen	DS	280.4 ± 41.80
Ferulic acid	DangGui	DG	1109 ± 105.0
5-hydroxymethyl furfuraldehyde	DiHuang	DH	2328 ± 90.34
Garlicin	DaSuan	DSS	1029 ± 53.87
Forskolin	MaHouQiaoRuiHua	MH	1.110 ± 0.1564
Panax notoginsenosides	SanQi	SQ	Exhibit about 20–25% inhibition rate at doses more than 2,500 μg/mL

### Results of generation 1 FSC analysis

**Figure 3:**
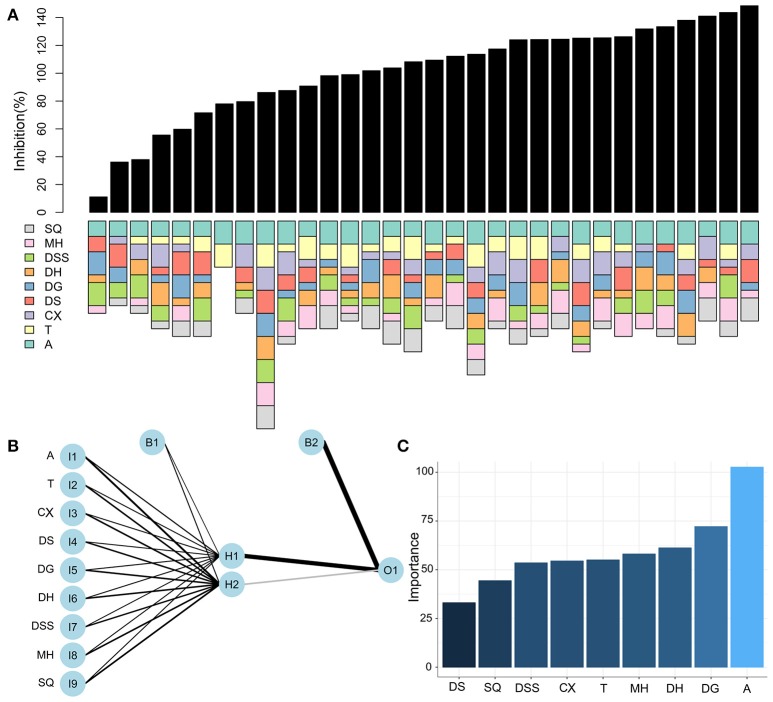
Results of FSC optimization in generation 1. **(A)** Efficacy and drug composite of the drug combinations in generation 1. The length of the colored bars underneath the inhibition data indicates the coded amount of dose for each drug. **(B)** The architecture of the neuron network used to train the 32 data points in Generation 1. **(C)** The relative importance of the drugs based on this model was calculated using Garson's algorithm. And the top five drugs were investigated in the following study. A, aspirin; T, ticagrelor; CX, ligustrazine from ChuanXiong; DS, Salvianolic acid B from DanShen; DG, ferulic acid from DangGui; DH, 5-hydroxymethyl furfuraldehyde from DiHuang; DSS, garlicin from DaSuan; MH, forskolin from MaHouQiaoRuiHua; SQ, panax notoginsenosides from Sanqi.

The FSC technique is based on a closed-loop feedback control process, aims to search for optimal drug combinations. In each generation, drug combinations are tested and analyzed, resulting in the development of fitted model. The analysis of the model allows for narrowing down the search space by eliminating ineffective or antagonistic drugs. The optimization process was initiated with 9 drugs based on their anti-platelet activities. For each drug, dose-response curve and IC50 value were fit by Hill equation (Chou, 2006, 2010) to identify dose levels used in the optimization process (Figure 2). 32 drug combinations were selected based on a designed matrix referred to as “orthogonal array composite design” (OACD). The efficacy and drug composite of the drug combinations were shown in Figure 3A. The obtained data were used to generate a two hidden node neuron network (Figure 3B). The weight of the parameters was indicated by the thickness of the line. The relative importance of the drugs toward anti-platelet activities based on this model was calculated using Garson's algorithm (Garson, 1991) (Figure 3C). And the 5 most dominant drugs (Aspirin, Ticagrelor, Ferulic acid, 5-hydroxymethyl furfuraldehyde, and Forskolin) were then selected for further optimization.

### Results of generation 2 FSC analysis

**Figure 4:**
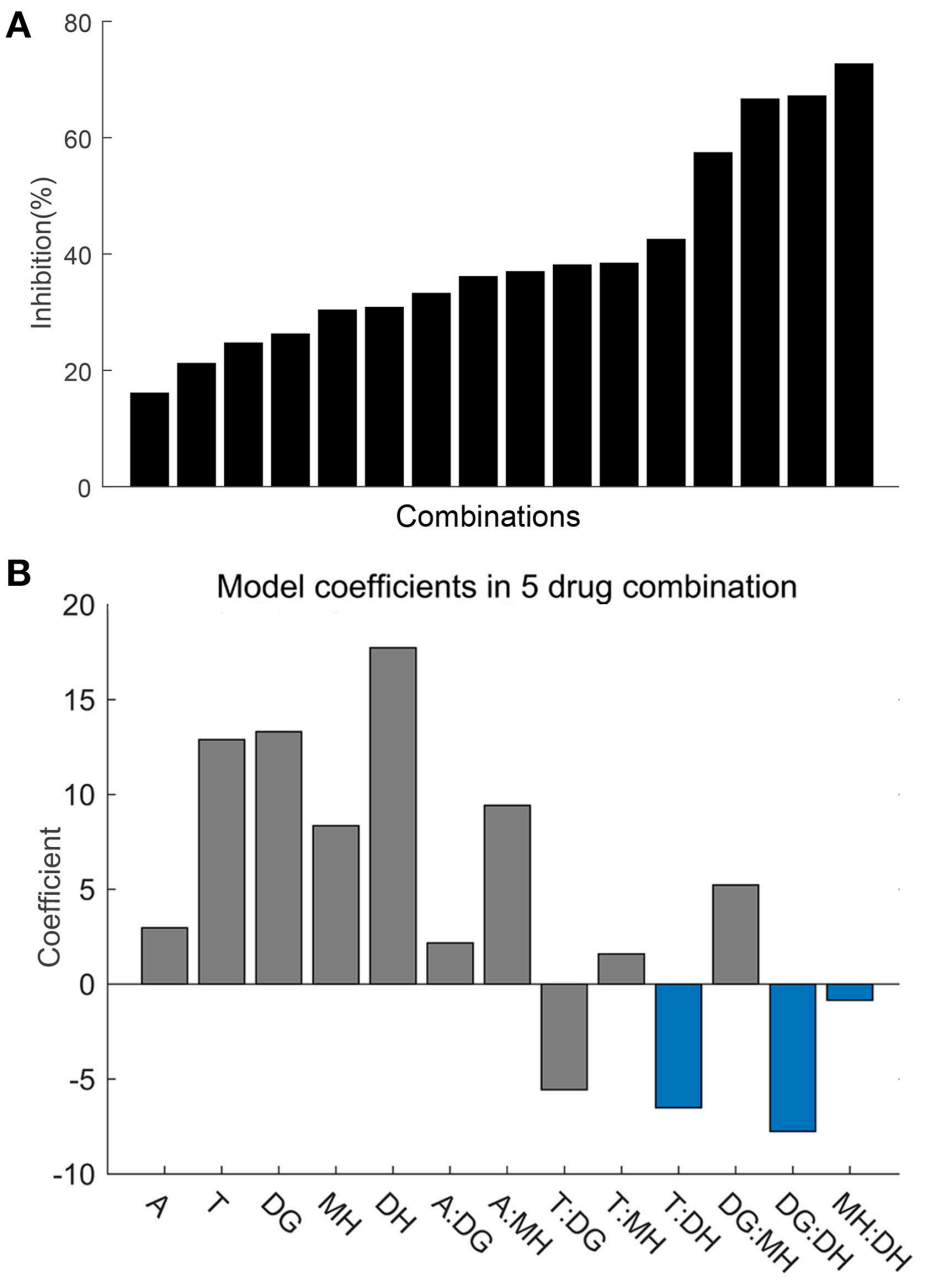
Results and data analysis of the 5-drug combination system. **(A)** The anti-platelet efficacies of the 16 drug combinations of 5-drug system investigated in generation 2. **(B)** Regression coefficients from the interaction model are provided based on the obtained data in **(A)**. Negative regression value indicates that increasing the dose of the drug reduces the inhibition ability of the combination. Thus the model allows for the elimination of the least active drug. The 5-hydroxymethyl furfuraldehyde related drug-drug interaction terms are all negative as highlighted by blue, indicating 5-hydroxymethyl furfuraldehyde was the least active drug in the combination. A, aspirin; T, ticagrelor; DG, ferulic acid; MH, forskolin; DH, 5-hydroxymethyl furfuraldehyde.

**Figure 5:**
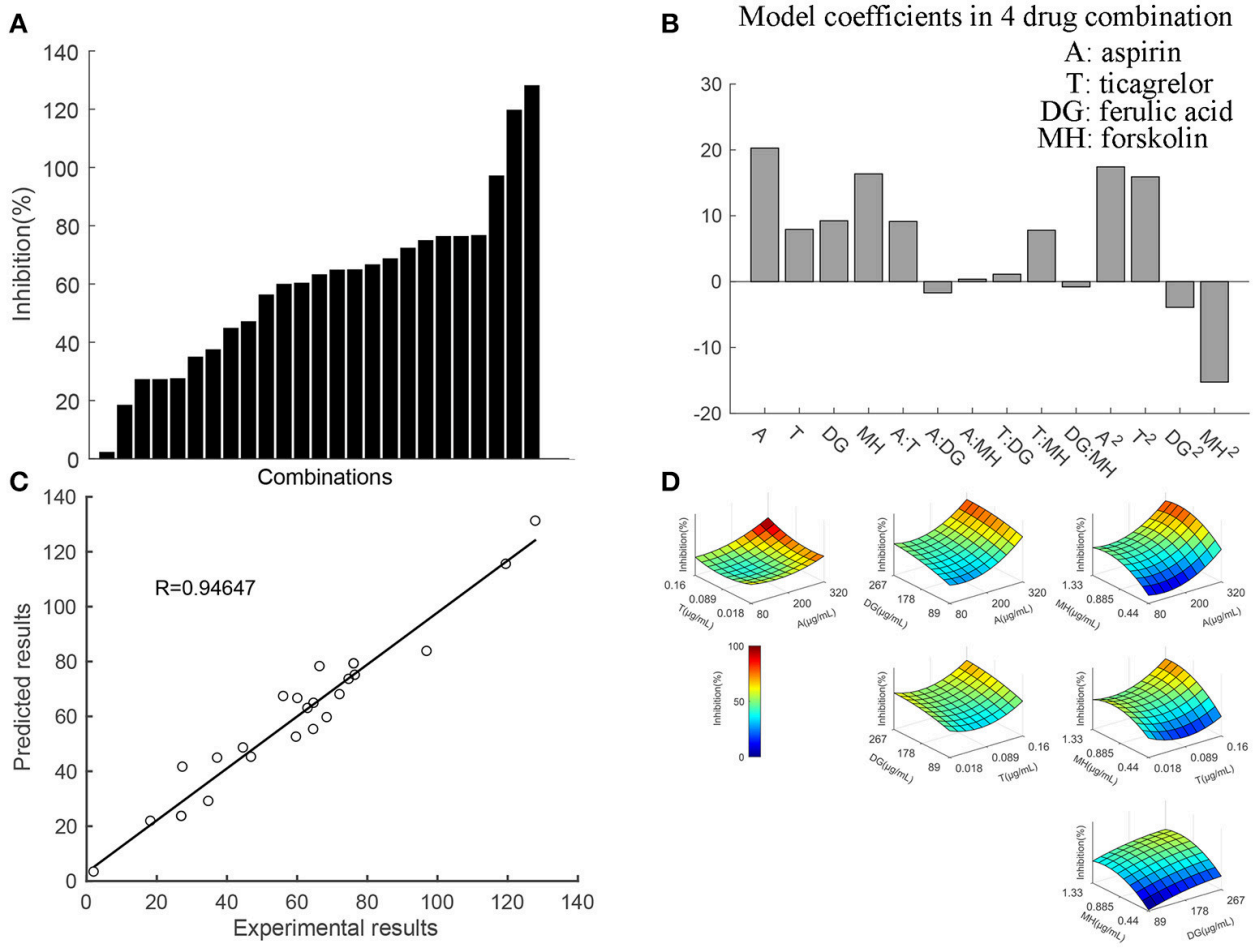
Results and data analysis of the 4-drug combination system. **(A)** The anti-platelet efficacies of the 25 drug combinations of 4-drug system investigated in generation 2. **(B)** Regression coefficients from the quadratic model were provided based on the obtained data. The positive values of A:T (aspirin:ticagrelor) and T:MH (ticagrelor:forskolin) indicated synergy between the two drug pairs, while negative values of A:DG (aspirin:ferulic acid) and DG:MH (ferulic acid:forskolin) suggested antagonism between them. **(C)** The correlation between experimentally tested efficacies (x-axis) and model predicted inhibition rate of the 25 drug combinations was shown. The correlation coefficient of the model is 0.94, which means the model can predict the experimental points with high fidelity. **(D)** The figure showed the model predicted anti-platelet efficacies when the concentrations of two drugs are held at constant while the concentrations of the other two drugs are varied.

In generation 2, firstly, combinations of the first 5 important drugs (aspirin, ticagrelor, ferulic acid, 5-hydroxymethyl, and forskolin) were designed base on OACD. The efficacies of the 5 drugs combination are provided in Figure 4A. These data points were used to generate an interaction regression model (Figure 4B). The model coefficients for each drug correspond to its impact on overall mixture efficacy, i.e., a positive regression value indicates that increasing the dose of that drug increases the inhibition effect on platelet. Therefore, the regression coefficients describing single linear drug effects and two-drug interaction terms are provided in this model. As indicated by the blue bar in Figure 4B, all the 5-hydroxymethyl furfuraldehyde (DH) related two drug interaction terms were negative, which suggested DH exhibited antagonism with other drugs in the combination. Therefore, DH was excluded from the system and further study of the 4-drug combination (aspirin, ticagrelor, ferulic acid, and forskolin) was conducted. The corresponding results of the 4-drug combination were presented in Figure 5A. The 25 data points obtained from the 4-drug combination were fitted with a quadratic regression model. Regression coefficient analysis presented in Figure 5B shows synergetic interactions between A:T (aspirin:ticagrelor) and T:MH (ticagrelor:forskolin), as well as antagonistic interactions between DG:MH (ferulic acid:forskolin) and A:DG (aspirin:ferulic acid). Figure 5C showed the correlation between experimentally tested efficacies (x-axis) and model predicted inhibition rate of the 25 drug combinations. The results suggested that the fidelity of the model in prediction the 25 experimental points was high since the correlation coefficient between experimental data and their corresponding predicted value was 0.94. Figure 5D showed the models in prediction of anti-platelet efficacies of drug pairs (i.e., when the concentrations of two drugs are held at constant while the concentrations of the other two drugs are varied).

### Results of drug interaction analysis

**Figure 6:**
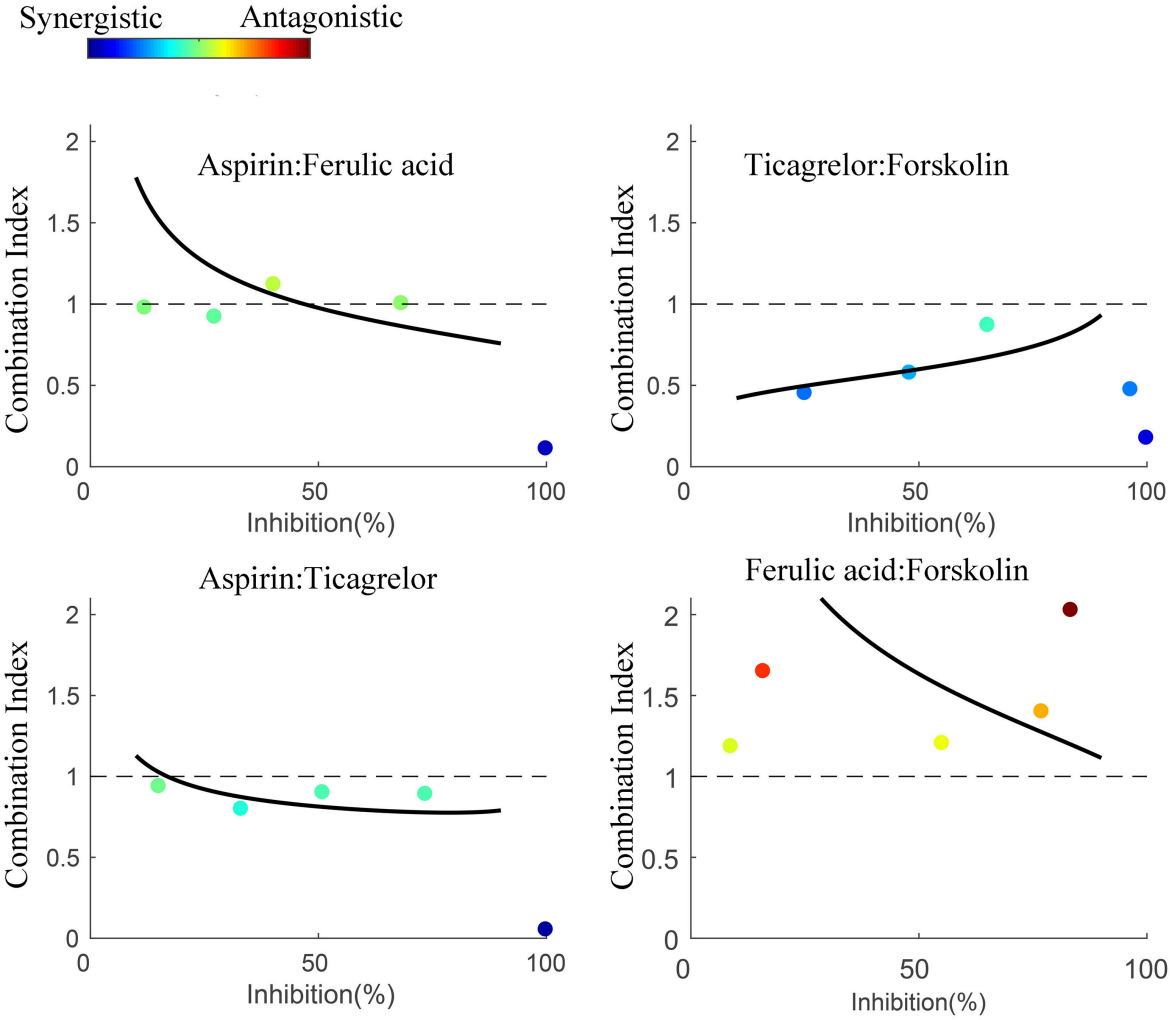
CI-Fa plot for the constant ratio experiment. The dots in the figure are the data points in the drug pair experiment with different concentrations. Combination Index value was calculated for each drug pairs as indicated by the color of the dots, red for antagonism and blue for synergistic. The results confirmed synergism between T:MH (ticagrelor:forskolin) and A:T (aspirin:ticagrelor), and antagonism between DG:MH (ferulic acid:forskolin) as predicted in generation 2. The interaction between A:DG (aspirin:ferulic acid) was found to be mainly additive effect.

In order to confirm the predicted interactions in generation 2, we performed an experiment set to evaluate the pairwise interactions between the 4 drugs. Totally, 4 drug pairs are studied with constant ratio experiment design, and the results of CI-Fa analysis applied to analyze the drug-drug interaction (Chou, 2006, 2010) were provided in Figure 6. The degree of the interaction between drug pairs is quantitatively measured using CI, with CI = 1 indicates additive effect, CI > 1 indicates antagonism and CI < 1 indicates synergism. As shown in Figure 6, the results suggested strong synergism between T:MH (ticagrelor:forskolin), weak synergism between A:T (aspirin:ticagrelor), antagonism between DG:MH (ferulic acid:forskolin) and mainly additive effect between A:DG (aspirin:ferulic acid).

## Discussion

Platelets play a critical role in thrombosis and hemostasis thus the pharmacological fine-tuning of the platelets is very important. In the present study, we tried to optimize possible combined use of DAPT and anti-platelet herbal medicines and clarify interactions among them. Lots of herbal medicines were reported to have anti-platelet activities (Liao, 2000). In the present study, 68 herb medicines with reported anti-platelet activities were summarized and listed according to the number of related references. The reported anti-platelet activities of some herb medicines could be confirmed in our experimental study. For example, as shown in Supplemental Table 1, ChuanXiong (Rhizoma Chuanxiong, *LigusticumwallichiiFranch*.) was the herb whose anti-platelet activities were most frequently reported. Our results of experimental *in vitro* ADP-induced PRP aggregation assay confirmed the anti-platelet activity of ligustrazine, the main active component of ChuanXiong. The IC50 value of ligustrazine in inhibiting platelet aggregation was determined to be 388.9 ± 21.69 μg/mL, which was consistent with previous reports (Shao et al., 2015; Wang et al., 2016). While, as shown in the results of the present study, the IC50 values of ligustrazine and other herbal components were rather high which suggested that the anti-platelet activities were weak or only moderate. Chemical modification might be necessary for possible development of these herbal components as new drugs in anti-platelet therapy strategy. In the present study, ticagrelor was used as the representative P2Y12 receptor antagonist in DAPT to study the interaction with herbal medicines. Both clopidogrel and ticagrelor are presently used in clinic. But, though clopidogrel had been used for a long time in clinic, its characteristics such as drug interactions, metabolism genetic polymorphisms, and variability in the antiplatelet response, limited its use. On the contrary, ticagrelor, as the first licensed perorally active and reversible P2Y12-receptor antagonist, was considered to be more predictable and more potent antiplatelet agent (May and Lincoff, 2012; Samoš et al., 2016).

Interestingly, in the present study, ferulic acid from DangGui and forskolin from MaoHouQiaoRuiHua were found to be able to form a 4-drug combination together with DAPT (aspirin and ticagrelor). DangGui (*Angelica sinensis*), a well-known TCM, was first documented in ShennongBencao Jing (Shennong's Materia Medica; 200–300 AD) and has been mainly used as a blood tonic agent (Chao and Lin, 2011). DangGui was widely used in many TCM herbal formulas such as DangguiBuxue Tang for treatment of menstrual disorders, DangguiLonghui Wan for treatment of constipation, DangguiNiantong Wan for treatment of rheumatoid arthritis, DangguiShaoyao San for treatment of Alzheimer's disease, and etc. (Mei et al., 1991; Xie et al., 2012; Fu et al., 2016). Ferulic acid was widely used as the marker compound for assessing the quality of DangGui and its products (Chao and Lin, 2011). Notably, ferulic acid sodium had been approved by State Drugs Administration of China as a drug for treatment of cardiovascular and cerebrovascular diseases (Wang and Ou-Yang, 2005). MaoHouQiaoRuiHua (*Coleus forskohlii*) is a popular traditional herb medicine used since ancient times for treatment of heart diseases, abdominal colic, and respiratory disorders, especially in India (Kanne et al., 2015). In China, there were also herbal medicine prescriptions containing MaoHouQiaoRuiHua such as QiaoruisuKoufuye (SFDA Approval Number Z10960005) and QiaoruisuJiaonang (SFDA Approval Number Z20113029) for the treatment of bronchitis. Forskolin is one of the main active components of MaoHouQiaoRuiHua (Schaneberg and Khan, 2003) and it is now in clinical trial for treatment of open angle glaucoma (Majeed et al., 2015). In all, DangGui (ferulic acid) and MaoHouQiaoRuiHua (forskolin) are both popularly used herbal medicines and possible interactions between them and DAPT are worthy to be clarified.

Weak synergism between aspirin and ticagrelor was found in the present study. The mechanisms of aspirin and ticagrelor in inhibiting platelet aggregation were inactivation of cyclooxygenase and antagonism of P2Y12 ADP receptor, respectively. Finding of synergism between aspirin and ticagrelor supported the popular use of DAPT as first-line standard antiplatelet medication in cardiovascular prevention. Consistently, previous reports also showed that aspirin had a demonstrable synergy of antithrombotic activity with P2Y12 antagonism (André et al., 2003; Schror, 2016). The synergistic anti-aggregating and anti-thrombotic effects of combination of aspirin and clopidofrel (ADP receptor antagonist) had been observed in several animal models (Herbert et al., 1998; Makkar et al., 1998) as well as in human samples (Moshfegh et al., 2000). Compared with monotherapy with aspirin, clopidogrel in combination with aspirin markedly inhibited ADP-mediated platelet aggregation. Simultaneous antagonism of thromboxane A2 by aspirin and ADP by clopidogrel resulted not only in inhibition of arachidonic acid and ADP-mediated platelet activation but also in a reduction of collagen- and thrombin-induced platelet activation (Moshfegh et al., 2000).

Other drug interactions found in our analysis such as synergism between T:MH (ticagrelor:forskolin) and antagonism between DG-MH (ferulic acid:forskolin) were also in accordance with the mechanisms of the drugs. Synergism between ticagrelor and forskolin was the strongest interaction observed in the 4-drug combination. The anti-platelet activities of forskolin from MH had been reported before and the mechanism was attributed to its unique character as a direct, rapid, and reversible activator of adenylyl cyclase (Adnot et al., 1982; Alasbahi and Melzig, 2012). Ticagrelor is a reversible antagonist of P2Y12 receptor, a 342 amino acid Gi-coupled receptor expressed on platelets. P2Y12 receptor is physiologically activated by ADP and could inhibit adenylyl cyclase to decrease cAMP level, resulting in platelet aggregation (Zhang et al., 2017). Previous report also showed that ticagrelor shifted the concentration-response curve of ADP to the right in inhibiting forskolin-induced cAMP formation (Hoffmann et al., 2014). Therefore, antagonism of P2Y12 receptor by ticagrelor might show synergistic effects with increase in cAMP level induced by forskolin. The mechanism of ferulic acid in inhibiting platelet aggregation had not been fully clarified. Reported effects of ferulic acid included influence on 5-HT release (Yin et al., 1980), TXA2/PGI2 balance (Xu et al., 1984), arachidonic acid metabolism (Xu et al., 1990), and activation of cAMP and cGMP signaling (Hong et al., 2016; Nadal et al., 2016). Effects of ferulic acid on cAMP might be the basis of antagonism between ferulic acid and forskolin (DG:MH). And, it is possible that effects of ferulic acid on TXA2/PGI2 balance and arachidonic acid metabolism resulted in additive effect with aspirin. Interactions between drugs found in the present study might deserve further study.

In all, FSC analysis was used in the present study to optimize dose strategy and clarify interactions in possible combined use of DAPT and herbal medicines. Our goal was to develop efficient and practical methods to optimize combinations of anti-platelet agents and provide insights into platelet signaling networks. Optimization of combined use in anti-platelet therapy is not only useful to avoid adverse reactions caused by interaction of herbal medicines with DAPT but might be also helpful to improve DAPT efficacy and safety in patients with cardiovascular diseases. Combined use of DAPT and anti-platelet herbal medicines could be a useful method to reach the sweet spot between ischemia and bleeding. A 4-drug combination was studied as an example. Results of the present study supported the use of FSC analysis in study of drug combinations in an-platelet therapy. FSC analysis might be helpful in designing personal anti-platelet therapy strategy considering the drugs which the patient needs to take at the same time. Furthermore, interactions between different drugs found in FSC analysis might provide useful information for research of signal cascades in platelet.

The merit of FSC application is that the scheme is purely based on biological system phenotype output, and does not rely on the intracelluar complex signaling network. Therefore, as long as the system readout can be accurately detected and the system input can be accurately controlled, FSC scheme can rapidly identify the most potent drug combinations from a large pool of drug candidates. Meanwhile, since the FSC mechanism focuses on the phenotype response, the technique does not make any assumption based on molecular signaling observation. Therefore, the effective drug combination identified from FSC scheme automatically ensures its potency. The successful demonstration of FSC scheme in this study serves as a benchmark for the combinatorial drug studies in other disease models.

## Author contributions

WL, Y-LL, M-TF, and Y-WZ: performed the experiments. XD, BH, and XL: conceived and designed the protocol. XD and XL: wrote the paper. All the authors reviewed and approved the submitted version of the paper.

### Conflict of interest statement

The authors declare that the research was conducted in the absence of any commercial or financial relationships that could be construed as a potential conflict of interest.
