# Supplementary material for: Application of Feedback System Control Optimization Technique in Combined Use of Dual Antiplatelet Therapy and Herbal Medicines
Source: Front Physiol. 2018 May 4;9:491. doi: 10.3389/fphys.2018.00491 (PMC5945866; doi:10.3389/fphys.2018.00491)
Supplement: Supplementary file 1 [file Image_1.PDF]

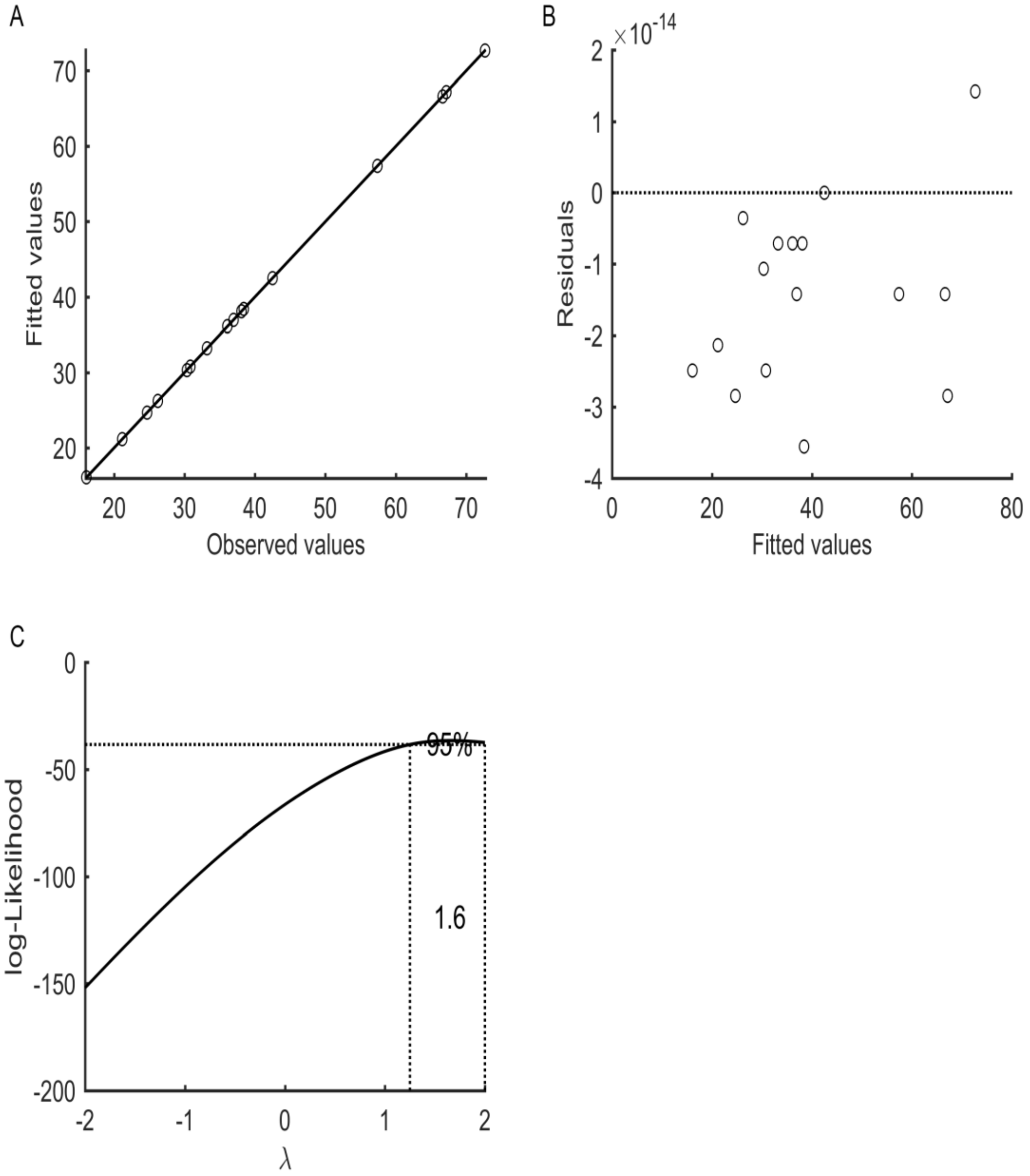

Supplemental Figure 1 Statistical analysis for interaction model used in generation 2. (A) The correlation between experimentally tested efficacies (x-axis) and model predicted inhibition efficacies of the 16 drug combinations was shown. (B) Residuals versus the fitted values plot for the model. (C) Log-likelihoods for the parameter λ of the Box-Cox power transformation for the linear model.
