# Supplementary material for: Application of Feedback System Control Optimization Technique in Combined Use of Dual Antiplatelet Therapy and Herbal Medicines
Source: Front Physiol. 2018 May 4;9:491. doi: 10.3389/fphys.2018.00491 (PMC5945866; doi:10.3389/fphys.2018.00491)
Supplement: Supplementary file 2 [file Image_2.PDF]

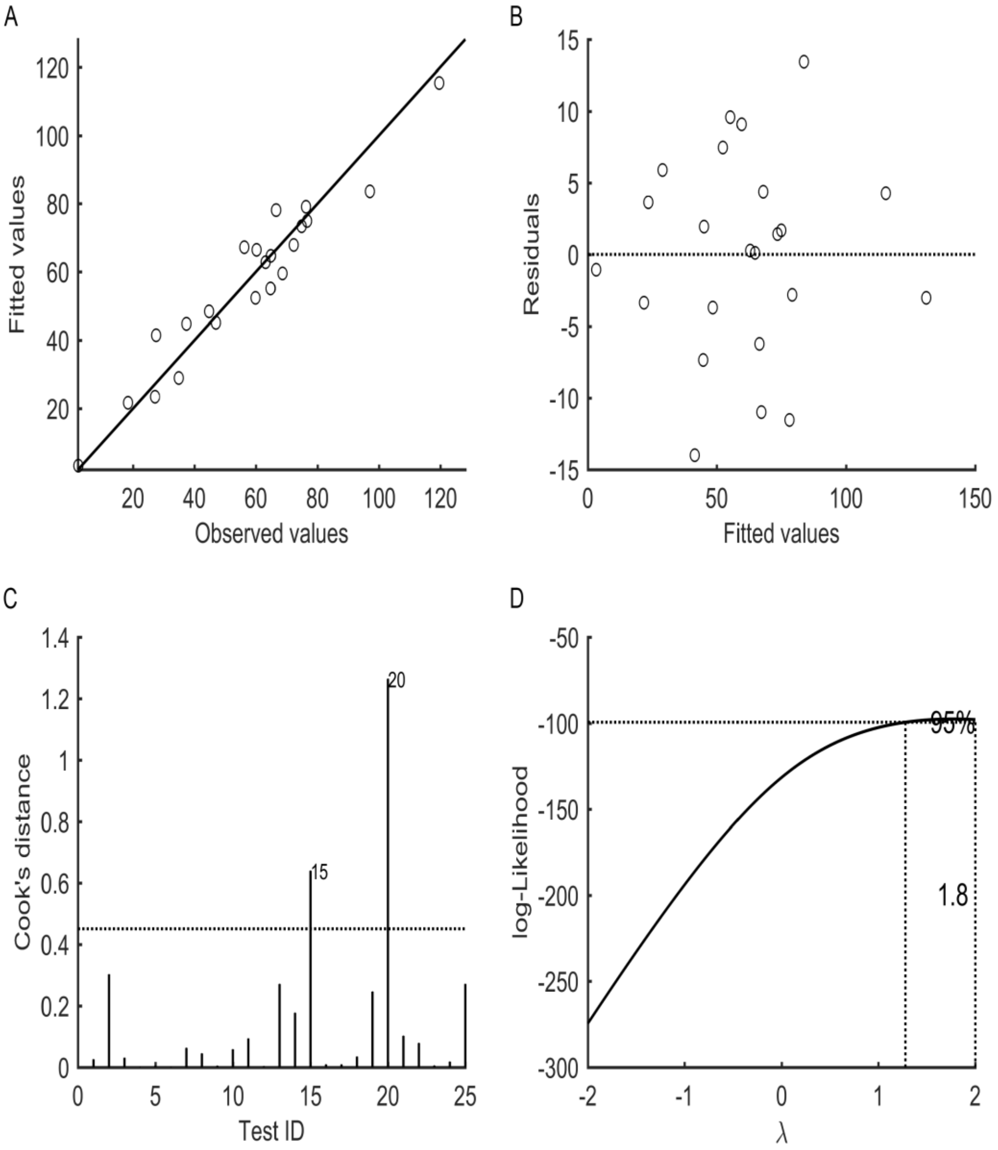

Supplemental Figure 2 Statistical analysis for quadratic model used in generation 2. (A) The correlation between experimentally tested efficacies (x-axis) and model predicted inhibition efficacies of the 25 drug combinations was shown. (B) Residuals versus the fitted values plot for the model. (C) Cook's distance plot for the model, combination#15 and #20 are considered as influential to the model. (D) Log-likelihoods for the parameter λ of the Box-Cox power transformation for the linear model.
