# Supplementary material for: Application of Feedback System Control Optimization Technique in Combined Use of Dual Antiplatelet Therapy and Herbal Medicines
Source: Front Physiol. 2018 May 4;9:491. doi: 10.3389/fphys.2018.00491 (PMC5945866; doi:10.3389/fphys.2018.00491)
Supplement: Supplementary file 3 [file Table_1.DOCX]

**Supplemental Table 1**. List of herbal medicines with reported anti-platelet activities.

| No. | Chinese name | English name | Latin name of herbs | Number of references |
| --- | --- | --- | --- | --- |
| 1 | ChuanXionɡ | Rhizoma Chuanxiong | *Ligusticum wallichii Franch.* | 23 |
| 2 | DanShen | Radix Salviae Miltiorrhizae | *Salvia miltiorrhiza Bunge.* | 20 |
| 3 | ChiShao | Radix Paeoniae Rubra | *Paeonia lactiflora Pall.* | 14 |
| 4 | DanɡGui | Radix Angelicae Sinensis | *Angelica sinensis （Oliv.） Diels* | 12 |
| 5 | HongHua | Flos Carthami | *Carthamus tinctorius L.* | 12 |
| 6 | SanQi | Notoginseng Radix Et Rhizoma | *Panax notoginseng (Burk.) F. H. Chen.* | 9 |
| 7 | JianɡXianɡ | Lignum Dalbergiae Odoriferae | *Dalbergia odorifera T. Chen* | 8 |
| 8 | HuanɡQi | Radix Astragali | *Astragalus membranaceus （Fisch.） Bge. var. mongholicus （Bge.）Hsiao* | 7 |
| 9 | DonɡChonɡXiaCao | Chinese Caterpillar Fungus | *Cordyceps sinensis （Berk.）Sacc.* | 7 |
| 10 | RenShen | Radix Ginseng | *Panax ginseng C. A. Mey.* | 6 |
| 11 | TaoRen | Semen Persicae | *Prunus persica （L.） Batsch* | 6 |
| 12 | DiHuanɡ | Radix Rehmanniae | *Rehmannia glutinosa Libosch.* | 5 |
| 13 | YiMuCao | Herba Leonuri | *Leonurus japonicus Houtt.* | 4 |
| 14 | XieBai | Bulbus Allii Macrostemonis | *Allium macrostemon Bge.* | 3 |
| 15 | MaoHouQiaoRuiHua | Coleus forskohlii | *Coleus forskohlii* *(Wild.) Briq.* | 3 |
| 16 | DaSuan | Garlic | *Allium sativum L.* | 2 |
| 17 | RuXianɡ | Frankincense | *Boswellia carterii Birdw. (B. sacra Fluek.)* | 2 |
| 18 | XueJie | Sanguis Draxonis | *Daemonorops draco Blume.* | 2 |
| 19 | WuZhuYu | Fructus Evodiae | *Evodia rutaecarpa (Juss.)Benth.* | 2 |
| 20 | YinXin | Folium Ginkgo | *Ginkgo biloba L．* | 2 |
| 21 | GanCao | Radix Glycyrrhizae | *Glycyrrhiza uralensis Fisch.* | 2 |
| 22 | BaJiaoHuiXianɡ | Fructus Anisi Stellati | *Illicium verum Hook. f.* | 2 |
| 23 | SanɡZhi | Ramulus Mori | *Morus alba L.* | 2 |
| 24 | MuDanPi | Cortes Moutan | *Paeonia suffruticosa Andr.* | 2 |
| 25 | DaXueTenɡ | Caulis Sargentodoxae | *Sargentodoxa cuneata (Oliv.) Rehd.et Wils.* | 2 |
| 26 | GouTenɡ | Ramulus Uncariae Cumuncis | *Uncaria rhynch0phylla (Miq.) Jacks.* | 2 |
| 27 | GanJianɡ | Dried Ginger Rhizoma | *Zingiber officinale Rosc.* | 2 |
| 28 | ZhiMu | Rhizoma Anemarrhenae | *Anemarrhena asphodeloides Bunge.* | 1 |
| 29 | LuoBuMaYe | Folium Apocyni Veneti | *Apocynum venetum L.* | 1 |
| 30 | BaiZhu | Rhizoma Atractylodis Macrocephalae | *Atractylodes macrocephala Koidz.* | 1 |
| 31 | CanɡShu | Rhizama Atractylodis | *Atractylodes lancea (Thunb.) DC.* | 1 |
| 32 | MuXianɡ | Radix Aucklandiae | *Aucklandia lappa Decne.* | 1 |
| 33 | DaYeGuanMen | Leaf of Manynerve Bauhinia | *Bauhinia pernervosa L. Chen* | 1 |
| 34 | ChaiHu | Radix Bupleuri | *Bupleurum chinense DC.* | 1 |
| 35 | ChenPi | Pericarpium Citri Reticulatae | *Citrus reticulata Blanco* | 1 |
| 36 | ShanHuanɡPi | Bark of Hollowed Wampee | *Clausena dentata （Willd.） Roem.* | 1 |
| 37 | YanHuSuo | Rhizoma Corydalis | *Corydalis yanhusuo W. T. Wang* | 1 |
| 38 | MoYao | Myrrh | *Commiphora myrrha Engl. (C. molmol Engl.)* | 1 |
| 39 | ShanZha | Fructus Crataegi | *Crataegus pinnatifida Bge.var.major N.E.Br.* | 1 |
| 40 | DanɡShen | Radix Codonopsis | *Codonopsis pilosula (Franch.) Nannf.* | 1 |
| 41 | XianɡFu | Rhizoma Cyperi | *Cyperus rotundus L.* | 1 |
| 42 | BaiXianPi | Cortex Dictamni | *Dictamnus dasycarpus Turcz.* | 1 |
| 43 | XianɡQinɡLan | Moldavian dragon's head | *Dracocephalum moldavica L.* | 1 |
| 44 | YinYanɡHuo | Herba Epimedii | *Epimedium brevicornum Maxim.* | 1 |
| 45 | LingZhi | Lucid Ganoderma | *Ganoderma lucidum* | 1 |
| 46 | ZhiZi | Fructus Gardeniae | *Gardenia jasminoides Ellis* | 1 |
| 47 | GanɡMei | Roughhaired Holly Root | *Ilex asprella (Hook. et Arn.) Champ. ex Benth.* | 1 |
| 48 | MaoDonɡQinɡ | Pubescent Holly Root | *Ilex pubescens Hook. et Arn.* | 1 |
| 49 | FenɡXianɡZhi | Resina Liquidambaris | *Liquidambar formosana Hance* | 1 |
| 50 | HouPo | Cortex Magnoliae Officinalis | *Magnolia officinalis Rehd. et Wils* | 1 |
| 51 | LianZiXin | Plumula Nelumbinis | *Nelumbo nucifera Gaertn.* | 1 |
| 52 | MaiDonɡ | Radix Ophiopogonis | *Ophiopogon japonicus （Thunb.）Ker-Gawl.* | 1 |
| 53 | QianHu | Radix Peucedani | *Peucedanum praeruptorum Dunn; Peucedanum decursivum Maxim.* | 1 |
| 54 | SonɡXianɡ | Colophony | *Pinus massoniana Lamb.* | 1 |
| 55 | LouYe | Bete Pepper | *Piper betle L* | 1 |
| 56 | HuJiao | Fructus Piperis | *Piper nigrum L.* | 1 |
| 57 | GeGen | Radix Puerariae | *Pueraria lobata （Willd.）Ohwi; Pueraria thomsonii Benth.* | 1 |
| 58 | ShiNanTenɡ | Piper Wallichii | *Piper wallichii （Miq.）Hand.-Mazz. var hupehense （DC.）Hand.-mazz.* | 1 |
| 59 | CanɡShu | Rhizoma Atractylodis | *Atractylodes lancea （Thunb.） DC.; Atractylodes chinensis （DC.） Koidz.* | 1 |
| 60 | QianCao | Radix Rubiae | *Rubia cordifolia L.* | 1 |
| 61 | FanɡFenɡ | Radix Saposhnikoviae | *Saposhnikovia divaricata (Turcz.) Schischk.* | 1 |
| 62 | WuWeiZi | Fructus Schisandrae Chinensis | *Schisandra chinensis （Turcz.）Baill.; Schisandra sphenanthera Rehd. et Wils.* | 1 |
| 63 | ShanLanɡDanɡ | Scopolia tangutica Maxim. | *Scopolia tangutica Maxim. [Anisodus tanguticus （maxim.） Pasch.]* | 1 |
| 64 | FanɡJi | Radix Stephaniae Tetrandrae | *Stephania tetrandra S. Moore* | 1 |
| 65 | ChenɡLiu | Tamarix sinensis Lour | *Tamarix chinensis Lour.* | 1 |
| 66 | JiLi | Fructus Tribuli | *Tribulus terrestris L.* | 1 |
| 67 | PuHuanɡ | Pollen Typhae | *Typha angustifolia L.; Typha orientalis Presl* | 1 |
| 68 | SuanZaoRen | Semen Ziziphi Spinosae | *Ziziphus jujuba Mill. var. spinosa （Bunge）Hu ex H.F.Chou* | 1 |
